# Distribution of mental health diagnoses in relation to sexual orientation and gender discontent in a late adolescent community population

**DOI:** 10.1186/s12888-025-07411-0

**Published:** 2025-10-13

**Authors:** Arne Gerdner, Therése Skoog, Sabina Kapetanovic, Emma Claesdotter-Knutsson, Susanna Askelöf, Anders Håkansson

**Affiliations:** 1 Department of Social work, Jönköping University, School of Health and Welfare, Jönköping, Sweden; 2 Department of Psychology, University of Gothenburg, Gothenburg, Sweden; 3 Department of Social and Behavioral Studies, University West, Trollhättan, Sweden; 4 Department of Clinical Sciences, Psychiatry, Lund University, Faculty of Medicine, Lund, Sweden

**Keywords:** Sexual orientation, Gender discontent, Psychiatric disorder, Substance use disorder, Addictive behavioral disorder, Prevalence, Adolescent, Community sample, Clinical interview

## Abstract

**Background:**

Few population studies have assessed the prevalence of various psychiatric disorders separately for sexual and gender minority groups, and none in an adolescent population. Given the increased mental health problems in sexual and gender minority groups combined, the present study aimed to estimate prevalence of mental disorders in separate sexual minority groups as well as gender discontent persons in community-recruited adolescents in Sweden.

**Methods:**

The present study is based on two waves (at 17 and 18 years of age) of a longitudinal cohort study (*N* = 949; 56% with female and 44% male legal gender, of which 1.7% reported gender discontent). Diagnostic data of 20 mental health disorders (psychiatric, substance use, and addictive behavior disorders) for each group of sexual orientation (asexual, homosexual, bisexual, and heterosexual) and gender discontent are reported, based on structured screening and subsequent diagnostic procedures.

**Results:**

The largest prevalence of mental health disorders was detected in homosexual girls and in bisexuals of both genders. Among asexual individuals, and among homosexual boys, none of the participants fulfilled the mental health disorders assessed. Gender discontent was associated with a moderately higher number of mental health disorders.

**Conclusion:**

Mental health services, both in specialized psychiatry and in community-based services targeting the mental health of adolescents may need to take into consideration the increased vulnerability for mental health disorders in bisexual individuals and in female homosexual individuals. Further research with larger samples is essential to better understand mental health in asexual individuals, young male homosexuals, and transgender or gender discontent populations.

**Supplementary Information:**

The online version contains supplementary material available at 10.1186/s12888-025-07411-0.

## Introduction

Recent decades have seen an increasing literature describing the mental health challenges of individuals referred to as sexual and gender minorities (i.e., among lesbian, gay, bisexual, queer and transgender populations and among others who do not identify sexually in line with what is considered the norm in society, LGBTQ+). Minority stress from victimization and discrimination as well as from various internalized stressors in LGBTQ+ populations is a commonly used framework for understanding poor mental health [1–3]. Early studies pointed at elevated suicidality among lesbian, gay, and bisexual (LGB) adolescents. Gibson’s report from 1989 [4] had a great impact on the debate at the time with the conclusion that gay adolescents were 2–3 times more likely to attempt suicide than other young people. These early studies were criticized by Savin-Williams [5] who argued that they did not represent the broad diversion among sexual minority youth, as they were based on selected samples and not on community samples. Savin-Williams also pointed out that only a small minority of persons with same-sex attraction actually identify themselves as LGB. Therefore, he concluded that studies based on self-identifying questions will not represent the whole group even when they are derived from a community sample. Another line of criticism was that the studies were not based on valid clinical interviews.

Although adolescents were in focus in Gibson’s report [4], relatively few studies on sexual minority (SM) mental health since then concerned adolescents, and none of these were based on clinical interviews. Adolescence is a challenging developmental period which includes finding one’s sexual identity. Addictive and other psychiatric disorders with early onset tend to peak in mid-adolescence [6]. Studies aiming to capture a possible development of poor mental health may therefore benefit from the inclusion of respondents in late adolescence. Likewise, late adolescence is also relevant due to more stability in sexual orientation than is the case in mid-adolescence. A recent longitudinal study on prevalence of sexual orientation [7] showed that although stability in sexual attraction pattern over the years dominated, there was also a significant fluidity especially in mid-adolescence.

In the years following, the studies broadened the perspective from suicidality in selected samples to epidemiologic studies of other emotional and substance use problems, mostly in community populations. Systematic reviews in the area focused mainly on suicidality, depression, anxiety disorders, and substance use disorders [8–15]. Most analyses in the early reviews failed to distinguish between different sexual minorities.

Three main methods have been used for the operationalization of sexual orientation [16]: (1) Self-identification, i.e., choosing a label such as gay, lesbian, bisexual, heterosexual etcetera that reflects “who you are”; (2) Self-reports on partners in sexual activities, usually same vs. opposite sex or both; and (3) Attraction patterns, using assessment scales. These methods vary in their ability to identify sexual orientation. A study by Black et al. [17] showed that men identifying as gay/bisexual were about half in number compared to those engaged in same-sex behavior. Variation in measurement sensitivity seems to be even more important in studies of young people developing their sexual identities. In a study on Norwegian adolescents, 16% reported same sex attraction, while half as many (8%) had made sexual contact with their same sex, yet only 3% identified as bisexual or gay/lesbian [18]. Based on a mixed-methods approach with a diverse sample of adolescents, Austin et al. [19] concluded that assessment of sexual attraction is a more valid and reliable way of quantifying sexual orientation among adolescents.

Similarly, in their methodological critique of the research thus far, Plöderl et al. [12] discussed operationalization of SM groups claiming that attraction pattern was more effective in terms of identifying persons compared to operationalization based on self-identification or sexual activities. They pointed out that this difference may also be selective in a way that causes bias in studies on SM and mental health, since those who had not yet fully “come out” as belonging to an SM group may be those suffering the most from minority stress with a greater risk of suicidality and other mental unhealth. Bagley and Tremblay [20] showed that homosexual individuals who had been sexually active in the past six months reported significantly lower levels of depression and fewer suicide attempts compared to sexually inactive homosexual individuals. It follows, according to Plöderl et al. [12], that studies using self-identity or sexual activity as the criteria will risk excluding precisely those persons who are most at risk of suicidality and mental disorders. To meet this challenge, we will follow the advice from Austin and colleagues [19] to operationalize sexual orientation based on attraction patterns. It seems especially appropriate in a study on adolescents, still in their formative years when sexual identities may sometimes not yet have stabilized [7] and many still have not had their sexual debut.

In the most extensive systematic review hitherto, Plöderl and Tremblay [13], examined 199 studies published up to 2014, focusing on the general population or specified populations, without presenting meta-analytic aggregates. Only 13% of the studies met high-quality criteria for representative populations and structured clinical interviews, with 27% presenting results by sex and sexual orientation. Transgender individuals were identified in only one study on smoking among young adults (0.3% of the population) but were later excluded from analyses [21]. Asexuals, mentioned in some studies, were either omitted or combined with other groups without separate analysis. Plöderl and Tremblay [13] highlighted four prevalent disorders across multiple studies: depression, suicidality, alcohol and drug use disorders, and anxiety-related conditions. Other disorders, such as eating disorders, schizophrenia, psychosis, PTSD, OCD, ADHD, and personality disorders, were less frequently studied or examined in singular studies.

A more recent systematic review analyzed mental health issues in sexual minorities based on 26 studies with 544,000 participants [15]. Lesbian/gay individuals had about twice the risk of mental disorders (depression, anxiety, alcohol use, suicidality) compared to heterosexuals, while bisexuals had a 1.5-5 times higher risk. Bisexuals also showed higher overall risks compared to lesbian/gay individuals. The studies varied in methods, with only 12 using diagnostic interviews, and differences in study quality affected the results. Studies using self-identified bisexuals showed larger differences compared to both heterosexuals and homosexuals than those based on attraction or sexual history.

A large cross-country WHO study [22] used structured interviews (CIDI) on adults to diagnose 17 specific disorders grouped into five types of disorders: mood, anxiety, and eating disorders, disruptive behavior (attention deficit disorder/ADD [with or without hyperactivity], intermittent explosive, and oppositional defiant/conduct disorder), and substance use disorders. Self-identity was used to define lesbian, gay, and bisexual individuals (LGB, 1.7%) as well as heterosexuals. Those who did not identify as LGB or as heterosexual, were omitted from the study (2.9%). Homosexuals of both genders had 2 to 4 higher risks (odds ratios) compared to heterosexuals concerning mood, anxiety, and eating disorders, but not concerning disruptive behavior or substance use disorders. Bisexuals had 3 to 5 times higher risks compared to heterosexuals concerning mood, anxiety, eating, and substance use disorders but not disruptive behavior disorders. Gender-separated analyses were conducted after combining homo- and bisexuals. Lesbian or bisexual women had 2 to 5 times increased risks compared to heterosexual women in mood, anxiety, eating, disruptive and substance use disorders with elevated risks for most specific disorders. Gay or bisexual men had about 3 times increased risk compared to heterosexual men for mood and anxiety disorders, with elevated risks concerning most specific disorders.

In a recent systematic review, Guz et al. [23] concluded that asexuality is a complex sexual orientation characterized by an individual’s low level of sexual attraction (or lack thereof) to other persons, independent of sex. Previous studies on asexuality are mostly qualitative, or quantitative studies based on non-probability samples, such as snowballing, convenience sampling, and members of internet communities. Consequently, Guz and colleagues suggest that asexuality should be included in epidemiological research on sexual orientation.

As mentioned, only one study in the extensive review by Plöderl and Tremblay [13] reported any transgender persons or persons in discontent with their attributed gender and these were excluded in the analyses. The lack of knowledge about transgenders’ or gender discontent persons’ epidemiology was confirmed in a scoping report [24] and in a review [25]. Since then, however, a recent Danish study [26] compared transgenders identified in hospital records or legal ID change (in total 0.06%) with those of the general population (> 15 years) on suicide and suicide attempts, showing 3.5 times higher rate of suicides and 7.7 on suicide attempts. Since gender dysphoria is a diagnostic category, all studies on the mental health of persons with gender dysphoria are based on clinical data or registry. A key component of gender dysphoria is the emotional dimension of being discontented with the gender attributed at birth [27]. Studies of gender discontented adolescents based on community samples concerning their mental health using structured diagnostic assessment are also still lacking.

### The situation for LGBTQ adolescents in Sweden

In recent years, several reforms to increase openness in society and to strengthen the equal rights of LGBTQ persons have been implemented in Sweden [28]. LGBTQ adolescents in Sweden generally experience a supportive environment, laws and policies regarding LGBTQ rights, which includes protections for sexual orientation and gender identity. Public attitudes towards LGBTQ individuals are largely positive, particularly in urban areas. However, LGBTQ adolescents may still face challenges, such as discrimination, bullying or isolation [29]. Sweden has comprehensive anti-discrimination laws. Schools are increasingly incorporating LGBTQ topics into their curricula, promoting inclusivity and awareness among all students. On 1 July 2025, a new law came into force (SFS no. 2024:238), giving persons the right to change their legal gender from the age of 16 years if it can be assumed that the person will live in this gender identity for the foreseeable future. Prior to this date, a change of legal gender could only be done from the age of 18 years and based on a transexual diagnosis.

In addition to various organizations and support groups aimed at helping LGBTQ youth, the healthcare system in Sweden is generally supportive of LGBTQ individuals, including adolescents. Sweden has a publicly funded healthcare system that aims to provide equal access to medical services for all citizens, including LGBTQ individuals. Adolescents can access healthcare without parental consent for certain services, which can be important for LGBTQ youth seeking help. Many regions offer specialized clinics and services focused on LGBTQ health. These may include sexual health clinics, mental health services, and support for gender identity and transition. Mental health services are increasingly recognizing the unique challenges faced by LGBTQ youth and efforts are being made to train healthcare providers in LGBTQ-specific issues.

### Gap of knowledge and the present study

Despite the large body of research, there is still a need for new studies. High-quality studies based on structured clinical interviews in community samples are still few. The disorders which are studied the most are mood, anxiety and substance use disorders, as well as suicidality [13, 15]. Eating disorders and disruptive behavioral disorders were also included in Gmelin et al. [22], while other mental health disorders have been represented only in few studies, e.g., psychotic syndrome and affective psychosis [13]. No study included behavioral disorders such as gambling or gaming disorders. Regarding SM groups, most studies concerned homo- and bisexuality [13, 15, 22]. Few identified asexuality as an SM group and asexual individuals were not analyzed separately. No representative study has reported on the mental health of the transgender/gender discontent group based on diagnostic data collected in community sample. There is still no high-quality study on SM adolescents’ mental health.

The present study aims to overcome these limitations by presenting prevalence of a broad spectrum of mental disorders, substance use disorders and behavioral disorders in four sexual orientation groups (asexuals, homosexuals, bisexuals, and heterosexuals) and gender discontented persons, using a community population in late adolescence.

## Methods

The present study is part of the Longitudinal Research on Development In Adolescence (LoRDIA), which started in 2013 by following 1885 adolescents from the age of 13 (grade 7, sub-cohort No. 1) or 12 (grade 6, sub-cohort No. 2) until the age of 18 (grade 12). LoRDIA was conducted in four small and middle-sized communities (< 40,000 inhabitants) in Sweden. The unemployment rate, annual income, educational level, and proportion of first-generation immigrants across the four municipalities were close to the national means [30].

Five data collection waves covered up to the age of 17 years with comprehensive questionnaires collected by the research team and assistants in classrooms. Among topics covered in the questionnaires were substance use and misuse, psychological health and ill-health, functional disability, social adaptation etcetera. In waves 3 to 5, questions on sexuality – attraction, behaviors, and experiences – were included, and in Wave 5 there was also a question on acceptance or discomfort with one’s gender. Wave 6 includes interviews with selected respondents.

### The two-phase design

As we notice from the reviews [8–15], there are some studies on SM adolescents’ emotional health, but we found no study on SM adolescents that used structured clinical interviews to assess psychiatric disorders. There are great methodological challenges in conducting population-based interview studies, e.g., the costs of required interviewers, and a strategy to handle expected data loss due to problems in contacting the persons or due to their unwillingness to participate in a lengthy interview. Possibly, it is an even greater challenge with adolescents. A two-phase design was proposed to make population interview studies more feasible [31], with a screening in Phase 1, directed towards the population to select those to be invited to structured interviews in Phase 2. The Phase 1 screening needs to have high sensitivity to include all relevant cases, and reasonably high specificity to exclude most of those who can be assumed to be non-cases. The interview instruments in Phase 2 need to have diagnostic validity based on established diagnostic criteria [32].

The present study is based on waves 5 and 6 from the LoRDIA program utilizing a two-phase design with screening in Wave 5, aiming to select those to be invited to structured clinical interviews in Wave 6. Wave 5 included screening instruments, specific for the disorders or group of disorders (Phase 1). The full information on screening instruments can be found in a previous publication [32]. They all had generous cut-offs, i.e.. high sensitivity, and were used to establishing who should be invited to interviews for clinical assessment. Wave 6 was conducted as telephone interviews using structured diagnostic instruments to establish whether selected interviewees met criteria for specific psychiatric disorders (Phase 2). The telephone interviews took place in the evenings, after school, conducted by 20 trained interviewers with professional skills in psychology or psychiatric nursing. In addition to professional merits, all 20 interviewers were trained by the authors AG and AH and were passed as qualified for the task. The training included a package of five web-based lectures on diagnostic theory and practices, the epidemiologic research interview and research ethics, eight realistic cases with exercises, detailed instructions for each of the instruments, and a filmed model interview. Finally, all trainees conducted training interviews, and these were reviewed in groups of three in web-conferences with AG and AH. Based on the data collected from the completed interviews in the data collection that followed, AG and AH decided together on the diagnostic outcomes for each interviewee.

Wave 5 questionnaires were collected in the autumns of 2017 and 2018, respectively, when the two cohorts were in their second year of upper secondary school (or 11th grade, in the Swedish 12-grade school system), about 17 years old. Wave 6 was carried out when adolescents were about 18 years old (or 12th grade).

### Participation and attrition

The first four waves of data collection had a high turn-out, with 93.4% participating at least once and 70–85% on each occasion. In Wave 5, about half (i.e., 50.3%) of the original study population participated. The analytical sample used here consisted of all 949 participants in Wave 5. These included 528 girls and 421 boys with a mean age of 17.0 years (SD = 0.44). Girls participated more than boys (56.9% vs. 43.9%, *p* < 0.001). The gender gap in response was handled by gender-separated analyses. Attrition in Wave 5 was further analyzed concerning demographic, socioeconomic, psychological health, and behavioral aspects, and presented in detail in our previous article [32]. Based on complete data-sets from taxation register, non-responders did not differ from responders in Wave 5 concerning foreign origin or living in relative poverty. There were also no differences concerning psychological wellbeing, psychosomatic problems, or negative consequences of drinking to intoxication as assessed in Wave 3. There was, however, some more attrition among those who scored higher on a delinquency scale (in Wave 3), which may result in some lower prevalence of externalizing behavioral problems. Considering the topic of this article, the previous Wave 5 non-response analysis was supplemented. Attrition in Wave 5 was unrelated to sexual orientation as well as to religious affiliation as assessed in Wave 3 [7]. Further, the internal dropout on questions on sexuality was very low (< 3%). This indicates that traditional norms and sexuality as being a sensitive area had little impact on data collection.

Altogether 758 adolescents (432 girls, 326 boys) scored above the cut-off on at least one of the screening indicators in Wave 5 and were selected to be interviewed with one or more of the assessment tools (see below), and 387 (237 girls, 150 boys) were interviewed in Wave 6, i.e., 51.1% of those selected. Non-response in Wave 6 was handled with estimations to adjust for attrition bias from the non-interviewed [32]. These estimations are presented in detail under the subheading Statistical Calculations, and the exact calculations are provided in an online supplement to facilitate transparency.

### Measures

**Legal gender** (male/female) is based on the personal ID-number given at birth or when given a residence permit, taken from tax records. It should be noted that the possibility for a person to change their legal gender from the age of 16 did not yet apply at this time, and no change of person ID-numbers within the LoRDIA population was reported from the taxation and population registry during these years.

**Gender discontent** was addressed with the question: “How do you feel in relation to your legally established gender? (The gender you have according to ID card and passport)”. Response alternatives were: (a) I am happy with my legal gender, (b) I have doubts about my legal gender, (c) I am not at all comfortable with my legal gender. In all, 941 (525 girls, 416 boys, 99% of both genders) responded to the question and of these 16 (1.7%) reported unease, including 15 who had doubts and one who was not at all comfortable with the legal gender. These 16 are in the following referred to as the gender discontent group. Ten of them had at birth been attributed a female legal gender and six a male legal gender.

**Sexual orientation** was addressed with the following double question on attraction: “Some are emotionally and sexually attracted to persons of *the opposite sex* and some to persons of *the same sex*. How do you feel? 1) To some person of the opposite sex? 2) To some person of the same sex?” Both questions were responded to by one of the following alternatives: (a) Not at all attracted emotionally and sexually, (b) Somewhat attracted emotionally and sexually, (c) Strongly attracted emotionally and sexually. There was a high response rate to both questions (97.3%). Based on these two questions, the following four categories of sexual orientation were created: The asexual group (*n* = 25; not at all attracted, neither to same nor to opposite sex); the homosexual group (*n* = 28; somewhat or strongly attracted to same sex, not at all to opposite sex); the heterosexual group (*n* = 746; somewhat or strongly attracted to opposite sex, not at all to same sex); and the bisexual group (*n* = 124; somewhat or strongly attracted both to same and opposite sex).

Note that all cutoffs on a scale are per definition simplifying. We chose these cutoffs of attraction in order to include adolescents who are still searching for their sexual identity, who may or may not yet have had a sexual debut with another, and who are supposedly more vulnerable to minority stress.

### Structured diagnostic assessment tools

The interviews were based on four instruments that assessed disorders based on DSM-5 or ICD-10 criteria:

**M.I.N.I.** (Mini International Neuropsychiatric Interview for DSM-5, version 7.0.1) [33] was used to assess several psychiatric conditions, i.e., depression, suicidality, mania, panic disorder, agoraphobia, social anxiety, OCD, PTSD, psychotic disorders, eating disorders, GAD, antisocial personality disorder (ASPD), ADHD and attention deficit disorder (ADD) in separate modules, all according to DSM-5. The M.I.N.I. also has modules addressing substance use disorders, but these modules were replaced by the instrument ADDIS (see below). Each module starts with two initial questions. If one of these is answered in the affirmative, they function as a key to open the module with all its questions, but if the initial questions are responded negatively, the interviewer goes on to the next module. M.I.N.I. was chosen since it is feasible for telephone interviews and was deemed suitable for epidemiologic study. The primary screening instrument used in Phase 1 to select for M.I.N.I. interviews was Mini Screen [34]. Among additional screening instruments for specific modules were the Psychosomatic Scale (PSP), a delinquency scale, and self-reported ADHD.

**ADDIS** (Alcohol and drug diagnosis instrument; adolescent version ADDIS-Ung) [35] was chosen to assess harmful use and dependence on 11 groups of substances (alcohol, sedatives, opioids, cannabinoids, cocaine, central stimulants, ecstasy, hallucinogens, solvents, other drugs, and mixed drugs). ADDIS is the Swedish version of the American SUDDS [36]. ADDIS can be used for assessment according to DSM-IV, DSM-5 as well as ICD-10. Here, the latter was chosen since the Swedish health care applies the concept of substance dependence based on ICD-10. The youth version (ADDIS-Ung) with some questions better adapted to adolescents was used. Primary screening instruments in Phase 1 for selection to ADDIS interview were AUDIT and DUDIT [37], with an additional scale on negative consequences of substance use.

**NODS** (National Opinion Research Center DSM-IV Screen for Gambling Problems) [38] was chosen to assess gambling problems. NODS can differentiate between no risk, risky gambling, problem gambling and pathological gambling (DSM-5) [39]. Screening in Phase 1 was based on two questions on having experiences of gambling for money. Having such experiences led to an invitation to interview.

**IGDS** (Internet Gaming Disorder Scale) [40]. Gaming disorder was introduced as a tentative diagnosis in the working process behind DSM-5 and later established as a manifest addictive diagnosis in the ICD-11. IGDS was used to assess gaming problems. IGDS differentiates between no risk, risky gaming, and pathological gaming [41]. Screening in Phase 1 was based on one question on time spent on video games, where spending 3–4 hours a day or more resulted in invitation to interview.

The quality – sensitivity, specificity, global and internal consistency, various forms of validity – of all four interview instruments, as well as all screening instruments and their cutoffs, are presented in detail in the previous article [32].

### Procedure

At the beginning of the research program, when students were 12 and 13 years old, they and their parents were informed about the research program, its confidentiality, and the voluntary basis of participation. The information letter to the parents was translated into their original 32 home languages other than Swedish and sent to both parents if living separately. The students were given the same information orally and in writing adapted to their age. Parents and students had the opportunity to decline consent for the students’ participation. From Wave 2, all names and personal ID numbers were replaced by four-digit code numbers in data files and in all collected questionnaires and interview forms. Only the administrator had the code key, thus the participants’ identities were unknown to the researchers.

Wave 6 depended on having participated in Wave 5. At the end of the Wave 5 questionnaire, information was provided about the planned interview and contact addresses (telephone and e-mail) were requested. Of the 949 Wave 5 participants, 95% provided the necessary information, which indicated preparedness for participating in interviews. Wave 5 included the Phase 1 screening instruments. The screening instruments on gambling and gaming were, however, not included in the questionnaires in Wave 5 for the first cohort. In the second cohort, all who participated in Wave 5 were screened also for these problems. But for the first cohort, the interviewers were instructed to screen for gambling and gaming on those who were contacted to be interviewed with M.I.N.I. or ADDIS, and if that screening then indicated that they scored above the cut-off, these responders would also be interviewed with NODS and IGDS, respectively. Concerning ethical approval, see Declarations.

### Statistical calculation

Several complementary indicators with generous cut-offs, i.e., with priority of high sensitivity, have been used in Phase 1 to include all cases relevant for interviewing. When estimating prevalence among all Wave 5 participants who responded on sexual orientation (*n* = 923) or on gender discontent (*n* = 941), we therefore assumed no diagnosis for those without screening indication of the addressed problem. We also assumed that among those selected for a particular interview (or a particular module in M.I.N.I.), the prevalence among those not interviewed (who were either not reached or declined participation) would be the same as among those interviewed, since all had screening outcomes above the cut-off. The prevalence (*P*) for a specific group (e.g., a sexual minority group) was therefore calculated using the following formula:

$$P\mathit\;\mathit=\mathit\;\mathit(D\mathit\;\mathit+\mathit\;\mathit(D\mathit\;\mathit/\mathit\;\mathit(D\mathit\;\mathit+\mathit\;ND\mathit)\mathit\;\mathit\ast\mathit\;NI\mathit)\mathit)\mathit\;\mathit/\mathit\;T$$

where *D* is the number in that group who met the diagnosis in Wave 6 interview, *ND* is the number with no diagnosis in the same interview, *NI* is the number of not interviewed but who should have been interviewed according to screening, and *T* is the total number of cases in the group within Wave 5.

Reinterpreted in text, we first took the observed number in a specific population who met the diagnosis in Wave 6 and added to that the estimate for the non-interviewees (the proportion who met the diagnosis of all interviewees multiplied by those who should have been interviewed in Wave 6 but were not). The observed diagnoses, summed up with the estimated diagnoses for the non-interviewed, were then divided by the total number of the specific group in Wave 5 to obtain the prevalence for that group. This was reported as a percentage (e.g., 0.01 was reported as 1%).

For gambling and gaming, screening for sub-cohort No. 1 had only been carried out for those interviewed with ADDIS or M.I.N.I. Therefore, a selection was first made of those in sub-cohort No. 2 who had not been interviewed with any of these instruments. The proportion with a gambling or gaming diagnosis, respectively, in this sub-cohort (*PD*_*2*_) was assumed to apply to the corresponding non-screened group in sub-cohort No. 1 (*NS*_*1*_). For gambling and gaming, the prevalence formula is therefore as follows:

$$P\;=\;(D\;+\;(D\;/\;(D\;+\;ND)\;\ast\;NI)\;+\;(PD_2\;\ast\;NS_1))\;/\;T$$

The added part (*PD*_2_ * *NS*_1_) is thus the estimate for those in sub-cohort No. 1 who were never screened, because they were not interviewed with ADDIS or M.I.N.I. For transparency, all calculations with these formulas can be found in the document published online parallel to this article.

The significance of differences between SM groups was tested against the Chi-2-distribution. For diagnoses and groups with positive estimates (> 0), the relative risks (RR) are calculated in comparison with heterosexuals of the same gender [as in 9, 11–12]. For the gender discontent group, RR was calculated in comparison with all others (non-GD). RR is calculated as the prevalence of the minority group divided by the prevalence of the comparison group.

The analyses presented on sexual orientation groups will be gender-separated. The attributed legal gender is questioned by gender discontented persons. Due to that and the small size of this group, gender discontented persons will in the following be reported as one group, not separately for legal genders.

## Results

The lifetime prevalence of 20 psychiatric disorders, including substance use disorders and addictive behavioral disorders, tested for statistical significance, are presented in Table 1 in relation to sexual orientation separately for females and males, and for the gender discontent group regardless of legal gender. RR is presented in case of positive prevalence estimates. Since no asexual girl or boy and no homosexual boy fulfilled any of the 20 diagnostic entities, these are not included in the table.

**Table 1:** Estimated prevalence (%) of mental health disorders (lifetime) in relation to sexual orientation and legal gender, as well as among gender discontented persons regardless of legal gender. Relative risks (RR) in comparison to heterosexuals of same gender, and compared with all others, respectively

	Female (legal gender), total *n* = 515 (a)					Male (legal), total *n* = 408 (a)			Gender discontent, total *n* = 941		
	Homosexual		Bisexual		Heterosexual	Bisexual		Heterosexual	Yes		No
n =	18		101		385	23		361	16		925
Diagnostic entity	%	RR^†^	%	RR^†^	%	%	RR^†^	%	%	RR^‡^	%
Suicidality	13.9^**^	5.15	9.3^**^	3.44	2.7	31.6^***^	7.90	4.0	9.7 n.s.	2.14	4.5
Depression	13.9 n.s.	0.70	34.9^**^	1.75	19.9	20.2^**^	3.88	5.2	0	-	4.5
Mania	0	-	6.9^***^	17.25	0.4	5.9 n.s.	2.95	2.0	0	-	1.8
Panic disorder	19.4 n.s.	2.34	20.5^***^	2.47	8.3	22.1^***^	17.00	1.3	27.1^***^	4.12	6.6
Agoraphobia	0.6 n.s.	0.55	6.5^**^	5.91	1.1	0	-	0.3	9.0^**^	7.14	1.3
Social anxiety	19.4^**^	5.24	11.8^**^	3.19	3.7	0	-	1.0	18.1^**^	5.28	3.4
Obsessive compulsive disorder (OCD)	2.8 n.s.	0.80	11.8^***^	3.37	3.5	23.7^***^	18.23	1.3	8.3 n.s.	2.02	4.1
Post traumatic stress disorder (PTSD)	0	-	2.5 n.s.	-	0	0	-	0	0	-	0.3
Generalized anxiety disorder (GAD)	0	-	2.6 n.s.	1.24	2.1	0	-	0.3	9.7^**^	7.46	1.3
Psychotic syndrome	0	-	6.4^**^	3.76	1.7	5.5 n.s.	2.62	2.1	0	-	2.8
Affective psychosis	0	-	1.3 n.s.	4.33	0.3	0	-	0	0	-	0.3
Bulimia	0	-	1.3 n.s.	1.86	0.7	0	-	0	0	-	0.3
Anorexia	6.9^***^	17.25	0	-	0.4	0	-	0	0	-	0.4
Antisocial personality disorder (ASPD)	0	-	1.3 n.s.	3.25	0.4	5.9^*^	7.38	0.8	9.0^***^	12.86	0.7
Attention deficit hyperactivity disorder (ADHD)	0	-	5.1^**^	5.29	0.7	11.9^***^	17.00	0.7	9.0^*^	6.92	1.3
Attention deficit disorder (ADD)	6.9 n.s.	4.60	1.3 n.s.	0.87	1.5	5.9^**^	14.75	0.4	0	-	1.2
Dependence/harmful use of alcohol	0	-	22.3 n.s.	1.46	15.3	0	-	11.9	0	-	14.3
Dependence/harmful use of drugs	0	-	4.4^**^	7.33	0.6	0	-	2.4	0	-	1.8
Gambling problem or disorder	0	-	2.2	-	0	7.0 n.s.	1.13	6.2	0	-	2.9
Gaming disorder	0	-	0	-	0	8.7 n.s.	2.18	4.0	0	-	1.9
Any anxiety disorder #	27.7 n.s.	1.39	24.3 n.s.	1.22	19.9	19.0^***^	7.04	2.7	22.9 n.s.	2.52	9.1
At least one of all 20 diagnoses above	53.3^*^	1.93	48.7^***^	1.76	27.6	29.9 n.s.	1.00	29.7	33.3 n.s.	1.10	30.4
No. of diagnoses (mean)	0.87		1.28		0.48	1.50		0.51	0.80		0.60

Significance of differences: ^*^p < 0.05; ^**^p < 0.01; ^***^p < 0.001

a) The columns for 11 female and 14 male asexuals, as well as for 10 male homosexuals are removed due to zero prevalence

^†^Compared with heterosexuals of same gender. ^‡^Compared with all others

^#^Any of panic anxiety, agoraphobia, social anxiety, OCD, PTSD or GAD

Homosexual girls had significantly higher prevalence of three disorders – suicidality, social anxiety, and anorexia – compared to heterosexual girls. RR indicated a more than five-fold increased risk for suicidality and social anxiety, and more than 17 times higher risk for anorexia. More than half had at least one disorder and the risk of that was about twice as high compared to heterosexual girls. For two other disorders – panic disorder and ADD – RR indicated a 2-4-fold increase, but which did not reach statistical significance.

Bisexuals were the group most affected by psychiatric problems among both genders. Bisexual girls had significantly higher prevalence compared to heterosexual girls in 10 of 20 disorders – suicidality, depression, mania, panic disorder, agoraphobia, social anxiety, OCD, psychotic syndrome, ADHD, and drug use disorder. For the most frequent disorder (depression, 34.9%) the RR for bisexuals was less than doubled since heterosexual girls also had high prevalence (19.9%). In all other disorders, RR varied from 2 to 17 times higher risk among bisexuals. The RR of having at least one of the disorders was 1.76, and the mean number of disorders among bisexual girls was higher than among heterosexual girls (1.28 vs. 0.48).

For 7 of the 20 disorders, bisexual boys had significantly higher prevalence compared to heterosexual boys, and from more than twofold up to 18 times higher relative risk in all these entities – suicidality, depression, panic disorder, OCD, ASPD, ADHD, and ADD. Any anxiety disorder (combined) was also significantly higher, but since the bisexual boys had less or none of other disorders (including alcohol use disorder), they had no increased risk of having at least one disorder (RR = 1.00). However, individuals among them who did have a disorder often had more than one. Therefore, the mean number of disorders was higher than among heterosexual boys (1.50 vs. 0.51).

The gender discontent group had significantly elevated prevalences compared with non-GD adolescents in six of the 20 disorders – panic disorder, agoraphobia, GAD, ASPD, ADHD, and ADD – but no prevalence in 12 of the remaining disorders. They did not have more than a marginally increased risk of having at least one disorder (RR = 1.10) and they had moderately higher mean number of disorders compared to non-GD (0.80 vs. 0.60).

The three most frequent disorders in homosexual girls were suicidality, social anxiety, and panic disorder. The three most frequent disorders in bisexual girls were depression, dependence/harmful use of alcohol, and panic disorder. For bisexual boys, the four most frequent were suicidality, obsessive-compulsive disorder (OCD), panic disorder and depression. For the gender discontent group, the two most frequent disorders were panic disorder and social anxiety.

## Discussion

As there are few studies describing mental health in separate groups of sexual and gender minorities, and as there is much less knowledge about this in adolescent populations, we wanted to estimate prevalences in data from a longitudinal population-based research study in Swedish adolescents. Most importantly, our study demonstrated that bisexuals of both genders and homosexual girls were the groups with the heaviest burden of mental health disorders, both in terms of the mean number of detected disorders, and in terms of relative risks of having the disorders, compared to heterosexuals of the same gender. In addition, the gender discontent group also had a higher mean number of mental health disorders compared to all others (non-GD) combined – but not as high as bisexuals of both genders and homosexual girls. However, except for bisexual girls, sexual minority groups were not over-represented with respect to substance use disorders, and in asexual girls and boys and in homosexual boys, no participants met the criteria of any of the mental disorders assessed.

### Increased risk of mental health disorders

The findings that bisexuals of both genders, and homosexual girls, had more mental disorders than heterosexuals of same gender, are in line with many previous studies. Early research, decades ago [4], pointed at a more than two-fold increase in suicidality. Later meta-analyses on mostly adults [1, 9] and on youths [11] came to similar conclusions. A recent meta-analysis showed even greater risks – about three times in homosexuals and about five times in bisexuals [15]. In our study, we found that bisexual girls had a more than three times increased risk of suicidality, homosexual girls more than five times, and bisexual boys nearly eight times increased risk for suicidality, compared to same-gender heterosexuals.

### Disorder-specific comparisons with literature

Our findings of specific disorders in homo- and bisexual individuals differ to some extent in relation to previous literature. Regarding affective and anxiety-related disorders, King and colleagues [9] reported at least 1.5 times increased risk in mood and anxiety disorders for LGB persons, and Marshal and colleagues [10] demonstrated similar results on depression. Both Wittgens and colleagues [15] and the cross-country study by Gmelin et al. [22] reported a two-fold increased risk in both mood and anxiety disorders for homosexuals and three times increase for bisexuals. In our study, these increased risks were seen for bisexuals of both genders, but not for homosexuals. The same pattern, although amplified (17 times), was found for mania in bisexual girls, whereas no such significant difference could be demonstrated in bisexual boys. Concerning anxiety disorders, we found increased risks for panic disorder and OCD in bisexuals of both genders, and for social anxiety among lesbian and bisexual girls, but we did not find any increased risk for GAD in these groups. When we collapsed all anxiety disorders into “any anxiety disorder”, as previously done in the cited reviews [1, 9, 13, 15], a significantly higher risk was found only in bisexual boys, whereas no significant differences were seen for homosexual and bisexual girls.

Regarding eating disorders, an increased prevalence is consistent with what has been described by Gmelin et al. [22] in lesbian and bisexual women, although in our study this could not be shown in other groups than in homosexual girls. In Gmelin’s study, lesbian and bisexual women had nearly five times higher risk of having any eating disorder, while the risk in gay and bisexual men was three times higher than same-sex heterosexuals.

The increased risk of antisocial personality disorder (ASPD), ADHD and ADD, seen in our study, can only partly be compared to previous studies. These disorders were not included in the meta-analyses, but Gmelin et al. [22] included them (or similar) in the disruptive behavior disorders. Lesbian/bisexual women (combined) had nearly five times more ADD (which included ADHD), and more than six times increase in defiant/conduct disorder (similar to ASPD), while gay/bisexual men (combined) had no increased risks compared to heterosexual men. In the current study, bisexual girls had three times more ASPD and five times more ADHD. Homosexual girls had more than four times increased risk (but non-significant) of ADD. Bisexual boys had seven times increased risk of ASPD and 14- and 17-times increased risk of ADD and ADHD, respectively. Thus, altogether, the increased prevalence of neuro-psychiatric conditions can be considered a relatively consistent finding so far, although future studies may need to harmonize the diagnostic categories assessed in the study. In our study, also, it should be borne in mind that the study of ASPD in young individuals constitutes a challenge and that prevalence figures may be altered later in adulthood, although this should not limit the value of the comparison made with non-SM subjects in this study.

In addition, bisexual girls in the current study had about a four times increased risk of psychotic syndrome and affective psychosis. Bisexual men, too, had more than a two-fold increased risk of psychotic syndrome but this did not reach significance due to small numbers. These psychotic problems were not studied in the cited studies.

### Few differences regarding substance use disorders

Somewhat unexpectedly, the present study did not demonstrate a clear increase in the risk of substance use disorder in sexual minority girls and boys. Except for the significant seven-fold increase in drug use disorders in bisexual girls, no significant increases compared to heterosexuals were demonstrated in alcohol or drug use disorders for homo- or bisexual girls, or boys.

Thus, this is in some contrast to previous literature. The meta-analysis of King and colleagues [9] reported an increased risk of both alcohol and drug dependence in homosexual and bisexual women compared to heterosexual women, and a risk increase, although somewhat smaller, also in homosexual and bisexual men. Their results were not presented separately for homo- and bisexuals. Looking at the more recent studies, both Wittgens et al. [15] and Gmelin et al. [22], showed a more than three-fold increase of any substance use disorder in homo- and bisexual women (combined) but not in homo- and bisexual men.

Altogether, while demonstrating a generally increased risk of other mental health disorders, substance use disorders could not be demonstrated here to be over-represented in SM in general. In the same setting as here, in relatively recent population surveys studies (2008–2015), sexual minority status was indeed associated with a relatively marked increase in substance use disorders, and interestingly, their link to mental health problems was stronger than in sexual majority individuals. Typical challenges associated with sexual minority stress were cited as risk factors of substance use [42]. Therefore, our findings from this adolescent population are in some contrast to both international and same-setting findings. It should be addressed in future research whether the risk increase may not have started to be apparent in an adolescent population in the age range studied here, and whether it develops later. Here, longer longitudinal studies in adolescents, following them over time into early adulthood, are needed.

### Homosexual boys and asexuals – need for larger studies

Only 10 homosexual boys were identified in the study, and the finding that they did not qualify for any diagnosis is at odds with other studies [9, 11, 12]. Likewise, only 25 female or male asexuals were identified, and in this group, also, none of them qualified for any of the diagnostic entities of mental disorders. In the latter group, previous literature does not allow for any comparisons. Altogether, for both homosexual boys and for asexual girls and boys, conclusions from this study are difficult to draw. Although asexuals constitute another SM, at least from the present findings it cannot be demonstrated that they are affected by a minority stress significant enough to cause mental health disorders. Whether this is the explanation, or whether the lack of diagnoses in the present study is mediated by other factors, remains to be studied. More and larger studies using diagnostic assessment in community populations are needed.

### No obvious risk increase in behavioral addictions

Regarding the behavioral addictive disorders studied in this paper, few conclusions can be drawn, as the prevalence was low in several of the SM groups. No increased risk was seen in female SM respondents, or in the gender discontent group, and the possible risk increase of gaming disorder in bisexual boys remains uncertain due to the limited number of affected individuals. Altogether, it remains to be understood whether non-substance-related addictive disorders may be driven by minority stress or other factors that are likely to affect the health of SM groups. It has been suggested in previous online survey studies that problematic gaming – but likely not problematic gambling for money – may be more common in SM [43], and it has been proposed that transgender individuals may theoretically be at a particularly high risk of gaming problems, for example due to online behaviors related to one’s minority status [43, 44]. However, later research in the field has failed to replicate this finding [45], while other data again support the idea that problem gambling also might be over-represented in sexual minorities [46]. While gambling and gaming disorder are diagnoses which are hitherto less often addressed in psychiatric comorbidity studies than substance use disorders are, future research will be needed to fully outline the role of online behaviors and related addictive behaviors in SM groups, and these may need to include other behavioral addictions than the two diagnoses assessed here.

### Mental health in the gender discontent group

This is, to our knowledge, the first study presenting the prevalence of mental health disorders based on diagnostic assessment in gender discontented persons recruited from a general population of adolescents. Although the study detected only 16 cases, they constitute 1.7% of the study population, which is more than the 0.3–0.6% found in previous adult studies [21, 26, 47] and in line with the 1.2–2.7% found in adolescent community populations according to a recent systematic review [47].

In absence of community population studies, the review of Haas et al. [48] discussed transgenders based on clinical studies. Alarmingly high suicide rates were reported in a clinical population of more than 2,000 persons who had undergone reassignment surgery [49]. The elevated suicidality was confirmed in the study by Erlangsen et al. [26], comparing registry data from hospitals. The gender discontent group in the present study had at least a two-fold increased risk of suicidality, but with these small numbers (low power), conclusive comparisons are difficult to make. They also had an increased risk of several anxiety disorders, resulting in more than a two-fold increased risk of having any anxiety disorder, although associations regarding OCD and any anxiety were not statistically significant. In addition, there was an increased risk of two more extrovert disorders – ASPD and ADHD (about 13 and 7 times, respectively). None in the gender discontent group had any mood disorder, psychotic disorder, eating disorder, substance use disorders, or other addictive behaviors. This is particularly noteworthy when it comes to alcohol use disorder and depression, since both are relatively frequent among non-GD. Therefore, there was hardly any increase in having at least one disorder in the gender discontent group, although these analyses are clearly limited by the low number of individuals in this group. Clearly, more and larger studies are needed in non-clinical community samples with gender discontent individuals. There is, however, a Dutch community study on 13-15-year-olds using a broader concept of “gender variant experience” (4% of the population), assessed with the Achenbach System of Empirically Based Assessment [50]. Independent of attributed gender at birth, these gender variant experiences were related to symptoms of anxiety, depression, somatic complaints, rule-breaking, aggressive behavior as well as attention, social, and thought problems. Although not directly comparable to diagnostic categories, these findings seem to be in line with our findings concerning anxiety and extrovert disorders, although not concerning depression.

### Methodological discussion

The LoRDIA program was conducted in four small and middle-sized communities, i.e., the type of communities where most Swedes live. One of these is a suburb to a large city. It is unknown to what extent the findings generalize to populations living in large cities.

One obvious disadvantage of the current study is the limited sample size, which gave small groups in asexuals, homosexual boys, and gender discontent. Power was low also for homosexual girls and bisexual boys but still, several significant results were found among them. Nevertheless, we consider it valuable to report on them as separate groups – also for meta-analytical studies in the future.

There has been ambiguity in how to handle asexuals in previous studies. Most studies did not attempt to identify them, as shown in the review by Plöderl and Tremblay [13]. This shortcoming seems to be consequential from treating sexual orientation as a one-dimensional concept with homosexuals at one end, heterosexuals at the opposite end, and bisexuals in between. In many operationalizations, asexuals are likely unidentified and often unintentionally collapsed with heterosexuals. Deselecting asexuals from the analyses, however, would keep asexual individuals invisible and would hide potential characteristics of this group specifically. The findings of this study show that although asexuals are limited in numbers, they distinguish both from other SM groups and from heterosexuals and should be treated as a separate group.

The method to identify SM groups through pattern of attraction instead of self-identity or history of partners was recommended by scholars, e.g. Austin et al. [19] and Plöderl et al. [12]. As shown, it is more sensitive in identifying homo- and bisexuals compared to the alternatives. The homo- and bisexuals in the current study make up 16.5% of the studied population, which can be compared to 4.5% in Wittgens et al. [15] with studies using various methods, and 1.7% in Gmelin et al. [22] who used a self-identifying question. We should consider that not all individuals reporting same-sex attraction will identify as anything other than heterosexual or feel a need to “come out” at any point in their lives. Therefore, operationalization based on attraction could be expected to differ from operationalization based on self-identity also in adults.

The method to identify gender discontented persons by a scale on satisfaction with attributed legal gender captures, as expected, more cases than using self-identifying questions on having a transsexual identity. It is here illustrated with the difference between prevalence of 1.7% in this study and the 0.3% found by Rath et al. [21]. Such self-identifying questions risk, according to Plöderl et al. [12], losing all who did not yet fully have “come out” as LGB or as transsexual and they may therefore be struggling even more with minority stress problems. The fact that RRs in our study often were relatively high compared to other studies might be an indication of such problems, which should be most relevant in an adolescent population. Our finding is nearly three times that of Erlangsen et al. [27] based on hospital data on related diagnoses and gender change of ID registry, which both are ultimate indicators at the end of a “coming-out-process”. In the setting where this study was conducted, Sweden, recent years have seen a large increase in the number of individuals seeking treatment for gender dysphoria [51], and given such changes in between countries and over time, the generalizability of data between highly diverse geographical settings can be limited.

The method used to estimate prevalence was part of our two-phase design, since it could handle attrition bias due to missing interviews. It was applied in our previous publication on prevalence and comorbidity [32]. The formulas for estimating prevalence were presented in the methods section, and the calculations based on these formulas are available online for transparency.

The alternative of leaving out all non-interviewed was not an option, since that would have led to great data loss and severely biased prevalences. Those interviewed (*n* = 387) were 40.1% of the analytical sample (*n* = 949). With the adjustment method, the prevalences of the total analytical sample could be estimated also based on all scorings below cutoff for diagnostic categories where an interview was not needed, and with aggregated estimates for diagnostic categories when interviews should have been conducted but were not.

We should also remind the reader about a limitation mentioned in Gerdner and Håkansson [32], that the study might have underestimated prevalence of ADHD and ASPD, since attrition was related to higher scores on delinquency in Wave 5 of LoRDIA, which defined our analytical population. Still, there is no reason to believe that this limitation affects the distribution of the assessed disorders between SM groups, since attrition in Wave 5 was unrelated to sexual orientation as assessed in Wave 3.

### Implications

The present study may have implications for future research and clinical practice. Although the study needs to be followed by future studies with the ability to include larger number of individuals in SM groups, the present findings again point to the importance for mental health services and preventive interventions to take sexual minorities in consideration.

Even though our study is small, the high rates of mental health disorders in bisexual adolescents indicate a need for awareness both in clinical context and social preventive efforts. Further studies should address whether the increased risk in bisexual individuals may be due to stigma or self-stigma related to ambiguous attraction to different genders, or to other factors. Likewise based on the findings here, mental health services including youth-oriented services may need to put effort in inclusive language, screening tools and interview techniques which are open to a range of emotional and sexual partnerships, beyond only addressing same-sex or opposite-sex partnerships. Increased awareness about the mental health of bisexual individuals, as well as homosexual individuals, is needed not only in specialized psychiatric contexts, but also in school-based and other child and adolescent mental health services.

Future studies need to include larger samples of asexual, homosexual, and gender discontented individuals, who were represented by relatively small samples of individuals in this study. Here, research challenges for the future include the inclusion of larger samples than here, deepening the understanding of mental health in these groups and enabling more narrow sub-group analyses than were possible here.

## Supplementary Information

Supplementary Material 1.

## Data Availability

The datasets generated and/or analyzed during the current study are not publicly available due to ethical regulations regarding the LoRDIA program. Swedish data protection laws restrict sharing of the data. According to these laws, the data can be made available for research projects with pre-defined and ethically approved research questions. Requests to use the LoRDIA data should be directed to the steering committee of the LoRDIA program, through the corresponding author. Data can be made available after the steering committee has reviewed the request and the Swedish Ethical Review Authority has approved the research questions.

## Abbreviations

ADDIS: Alkohol Drog Diagnos InStrument
ADD: Attention Deficit Disorder
ADHD: Attention Deficit Hyperactivity Disorder
AUDIT: Alcohol Use Disorders Identification Test
ASPD: AntiSocial Personality Disorder
CIDI: The Composite International Diagnostic Interview
DSM-5: Diagnostic and Statistical Manual of Mental Disorders (fifth edition)
DSM-IV: Diagnostic and Statistical Manual of Mental Disorders (fourth edition)
DUDIT: Drug Use Disorders Identification Test
GAD: Generalized Anxiety Disorder
ICD-10: International Statistical Classification of Diseases and Related Health Problems (tenth revision)
ID: Identification
IGDS: Internet Gaming Disorder Scale
LGB: Lesbian, Gay, Bisexual
LGBTQ: Lesbian, Gay, Bisexual, Transgender, Queer
LGBTQ+: See above. The + refers to others who do not identify sexually in line with what is considered the norm in society
LoRDIA: Longitudinal Research on Development In Adolescence
NODS: National Opinion Research Center DSM‐IV Screen for Gambling Problems
Non-GD: Non-Gender-Discontent
M.I.N.I.: Mini International Neuropsychiatric Interview
OCD: Obsessive Compulsive Disorder
PTSD: Post Traumatic Stress Disorder
RR: Relative Risk
SD: Standard Deviation
SM: Sexual Minority
SUDDS-IV: Substance Use Disorder Diagnostic Schedule (fourth version)
