# Supplementary material for: Distribution of mental health diagnoses in relation to sexual orientation and gender discontent in a late adolescent community population
Source: BMC Psychiatry. 2025 Oct 13;25:972. doi: 10.1186/s12888-025-07411-0 (PMC12516857; doi:10.1186/s12888-025-07411-0)
Supplement: Supplementary file 1 — Supplementary Material 1. [file 12888_2025_7411_MOESM1_ESM.docx]

# Estimations of prevalence of psychiatric disorders for sexual or gender minority groups

Supplement to the article “Distribution of mental health diagnoses in relation to sexual orientation and gender discontent in a late adolescent community population”, *BMC Psychiatry*, 2025.

The study utilizes a two-phase design with the use of a screening in phase 1, aiming to select those to be invited to structured clinical interviews in phase 2. This supplement provides the calculations behind Table 1 in that article

We assumed no diagnosis for those without screening indication of the addressed problem, We also assumed that among those selected for a particular interview (or a particular module in M.I.N.I.), the prevalence among those not interviewed (who were either not reached or declined participation) would be the same as among those interviewed, since all had screening outcomes above the cut-off.

The prevalence (*P*) for a specific sexual orientation group was calculated using the following formula:

*P = (D + (D / (D + ND) * NI)) / T*

where *D* is the number in that group who met the diagnosis in Wave 6 interview, *ND* is the number with no diagnosis in the same interview, *NI* is the number of not interviewed but who should have been interviewed according to screening, and *T* is the total number of cases in the specific group within the analytical sample (Wave 5).

For gambling and gaming, screening for sub-cohort No. 1 had only been carried out for those interviewed with ADDIS or M.I.N.I. Therefore, a selection was first made of those in sub-cohort No. 2 who had not been interviewed with any of these two instruments. For gambling and gaming, the prevalence formula is as follows:

*P = (D + (D / (D + ND) * NI) + (PD_2_ * NS_1_)) / T*

Where *PD_2_* is the proportion with a diagnosis in sub-cohort No.2, assumed to apply to the corresponding non-screened group in sub-cohort No. 1 (*NS_1_*).

The added part (*PD_2_ * NS_1_*) is the estimate for those in sub-cohort No. 1 who were never screened, because they were not interviewed with ADDIS or M.I.N.I.

The first column presents the calculations of prevalence, and the second column provides the proportion of those who were not interviewed but should have been based on screening.

**Estimation of prevalence Proportion of selected**

**but not interviewed**

**Depression**

Homosexual women (2 + (2 / (2 + 10) * 3)) / 18 = 13.9 % 3 / 18 = 0.166

Bisexual women (25 + (25 / (25 + 36) * 25)) / 101 = 34.9 % 25 / 101 = 0.247

Bisexual men (3 + (3 / (3 + 8) * 6)) / 23 = 20.2 % 6 / 23 = 0.260

Heterosexual women (45 + (45 / (45 + 114) * 106)) / 385 = 19.4 % 106 / 385 = 0.275

Heterosexual men (13 + (13 / (13 + 121) * 60)) / 361 = 5.2 % 60 / 361 = 0.166

**Suicidality**

Homosexual women (2 + (2 / (2 + 10) * 3)) / 18 = 13.9 % 3 / 18 = 0.166

Bisexual women (7 + (7 / (7 + 54) * 21)) / 101 = 9.3 % 21 / 101 = 0.207

Bisexual men (5 + (5 / (5 + 6) * 5)) / 23 = 31.6 % 5 / 23 = 0.217

Heterosexual women (7 + (7 /(7 + 152) * 73)) / 385 = 2.7 % 73 / 385 = 0.189

Heterosexual men (10 + (10 / (10 + 124) * 48)) / 361 = 4.0 % 48 / 361 = 0.132

Gender discontent (1+ (1 / (1 + 8) * 5)) / 16 = 9.7 % 5 / 16 = 0.312

**Mania**

Bisexual women (5 + (5 / (5 + 56) * 24)) / 101 = 6.9 % 24 / 101 = 0.237

Bisexual men (1 + (1 / (1 + 10) * 4)) / 23 = 5.9 % 4 / 23 = 0.173

Heterosexual women (1 + (1 / (1 + 158) * 86)) / 385 = 0.4 % 86 / 385 = 0.223

Heterosexual men (5 + (5 / (5 + 129) * 57)) / 361 = 2.0 % 57 / 361 = 0.157

**Panic syndrome**

Homosexual women (3 + (3 / (3 + 9) * 2)) / 18 = 19.4 % 2 / 18 = 0.111

Bisexual women (16 + (16 / (16 + 45) * 18)) / 101 = 20.5 % 18 / 101 = 0.178

Bisexual men (4 + (4 / (4 + 7) * 3)) / 23 = 22.1% 3 / 23 = 0.130

Heterosexual women (23 + (23 / (23 + 136) * 61)) / 385 = 8.3 % 61 / 385 = 0.158

Heterosexual men (4 + (4 / (4 + 130) * 28)) / 361 = 1.3 % 28 / 361 = 0.077

Gender discontent (3 + (3 / (3 + 6) * 4)) / 16 = 27.1 % 4 /16 = 0.250

**Agora phobia**

Homosexual women (1 + (1 / (1 + 11) * 2)) / 18 = 0.6 % 2 / 18 = 0.111

Bisexual women (5 + (5 / (5 + 56) * 19)) / 101 = 6.5 % 19 / 101 = 0.188

Heterosexual women (3 + (3 / (3 + 156) * 61)) / 385 = 1.1 % 61 / 385 = 0.158

Heterosexual men (1 + (1 / (1 + 133) * 28)) / 361 = 0.3 % 28 / 361 = 0.077

Gender discontent (1+ (1 / (1 + 8) * 4)) / 16 = 9.0 % 4 / 16 = 0.250

**Social anxiety**

Homosexual women (3 + (3 / (3 + 9) * 2)) / 18 = 19.4 % 2 / 18 = 0.111

Bisexual women (9 + (9 / (9 + 52) * 20)) / 101 = 11.8 % 20 / 101 = 0.198

Heterosexual women (10 + (10 / (10 + 149) * 66)) / 385 = 3.7 % 66 / 385 = 0.171

Heterosexual men (3 + (3 / (3 + 131) * 31)) / 361 = 1.0 % 31 / 361 = 0.085

Gender discontent (2+ (2 / (2 + 7) * 4)) / 16 = 18.1 % 4 / 16 = 0.250

**Obsessive compulsive disorder (OCD)**

Homosexual women (2 + (2 / (2 + 10) * 3)) / 18 = 13.9 % 3 / 18 = 0.166

Bisexual women (10 + (10 / (10 + 51) * 12)) / 101 = 11.8 % 12 / 101 = 0.118

Bisexual men (4 + (4 / (4 + 7) * 4)) / 23 = 23.7 % 4 / 23 = 0.173

Heterosexual women (10 + (10 / (10 + 149) * 55)) / 385 = 3.5 % 55 / 385 = 0.142

Heterosexual men (4 + (4 / (4 + 130) * 26)) / 361 = 1.3 % 26 / 361 = 0.072

Gender discontent (1 + (1 / (1 + 8) * 3)) / 16= 8.3 % 3 / 16 = 0.187

**Posttraumatic stress disorder (PTSD)**

Bisexual women (2 + (2 / (2 + 59) * 16)) / 101 = 2.5 % 16 / 101 = 0.158

**Psychotic syndrome**

Bisexual women (5 + (5 / (5 + 55) * 17)) / 101 = 6.4 % 17 / 101 = 0.168

Bisexual men (1 + (1 / (1 + 10) * 3)) / 23 = 5.5 % 3 / 23 = 0.130

Heterosexual women (5 + (5 / (5 + 154) * 55)) / 385 = 1.7 % 55 / 385 = 0.142

Heterosexual men (6 + (6 / (6 + 128) * 33)) / 361 = 2.1 % 33 / 361 = 0.091

**Affective psychosis**

Bisexual women (1 + (1 / (1 + 60) * 17)) / 101 = 1.3 % 17 / 101 = 0.168

Heterosexual women (1 + (1 / (1 + 158) * 55)) / 385 = 0.3 % 55 / 385 = 0.142

**Anorexia nervosa**

Homosexual women (1 + (1 / (1 + 11) * 3)) / 18 = 6.9 % 3 / 18 = 0.166

Heterosexual women (1 + (1 / (1 + 158) * 61)) / 385 = 0.4 % 61 / 385 = 0.158

**Bulimia**

Bisexual women (1 + (1 / (1 + 60) * 17)) / 101 = 1.3 % 17 / 101 = 0.168

Heterosexual women (2 + (2 / (2 + 157) * 54)) / 385 = 0.7 % 54 / 385 = 0.140

**Generalized anxiety disorder (GAD)**

Bisexual women (2 + (2 / (2 + 59) * 18)) / 101 = 2.6 % 18 / 101 = 0.178

Heterosexual women (6 + (6 / (6 + 153) * 59)) / 385 = 2.1 % 59 / 385 = 0.153

Heterosexual men (1 + (1 / (1 + 133) * 26)) / 361 = 0.3 % 26 / 361 = 0.072

Gender discontent (1 + (1 / (1 + 8) * 5)) / 16 = 9.7 % 5 / 16 = 0.312

**Antisocial personality disorder (ASPD)**

Bisexual women (1 + (1 / (1 + 60) * 19)) / 101 = 1.3 % 19 / 101 = 0.188

Bisexual men (1 + (1 / (1 + 10) * 4)) / 23 = 5.9 % 4 / 23 = 0.173

Heterosexual women (1 + (1 / (1 + 158) * 69)) / 385 = 0.4 % 69 / 385 = 0.199

Heterosexual men (2 + (2 / (2 + 132) * 57)) / 361 = 0.8 % 57 / 361 = 0.157

**Attention deficit hyperactivity disorder (ADHD)**

Bisexual women (4 + (4 / (4 + 57) * 17)) / 101 = 5.1% 17 / 101 = 0.168

Bisexual men (2 + (2 / (2 + 9) * 4)) / 23 = 11.9 % 4 / 23 = 0.173

Heterosexual women (2 + (2 / (2 + 157) * 64)) / 385 = 0.7 % 64 / 385 = 0.166

Heterosexual men (2 + (2 / (2 + 132) * 43)) / 361 = 0.7 % 43 / 361 = 0.119

Gender discontent (1 + (1 / (1 + 8) * 4)) / 16 = 9.0 % 4 / 16 = 0.250

**Attention deficit disorder (ADD)**

Homosexual women (1 + (1 / (1 + 11) * 3)) / 18 = 6.9 % 3 / 18 = 0.166

Bisexual women (1 + (1 / (1 + 60) * 17)) / 101 = 1.3 % 17 / 101 = 0.168

Bisexual men (1 + (1 / (1 + 10) * 4)) / 23 = 5.9 % 4 / 23 = 0.173

Heterosexual women (4 + (4 / (4 + 155) * 64)) / 385 = 1.5 % 64 / 385 = 0.166

Heterosexual men (1 + (1 / (1 + 133) * 43)) / 361 = 0.4 % 43 / 361 = 0.119

**Harmful use or dependence on alcohol**

Bisexual women (16 + (16 / (16 + 11) * 11)) / 101 = 22.3 % 11 / 101 = 0.108

Heterosexual women (35 + (35 / (35 + 34) * 47)) / 385 = 15.3 % 47 / 385 = 0.122

Heterosexual men (22 + (22 / (22 + 33) * 52)) / 361 = 11.9 % 52 / 361 = 0.144

**Harmful use or dependence on drugs**

Bisexual women (4 + (4 / (4 + 24) * 3)) / 101 = 4.4 % 3 / 101 = 0.029

Heterosexual women (2 + (2 / (2 + 69) * 11)) / 385 = 0.6 % 11 / 385 = 0.028

Heterosexual men (7 + (7 / (7 + 49) * 12)) / 361 = 2.4 % 12 / 361 = 0.033

**Gambling problem/pathological gambling**

Bisexual women (1 + (1 / (1 + 19) * 4) *+* (0 * 26)) / 54 = 2.2 % 4 / 54 = 0.074

Bisexual men (1 + (1 / (1 + 4) * 3) *+* (0 * 3)) / 23 = 7.0 % 3 / 23 = 0.130

Heterosexual men (10 + (10 / (10 + 63) * 90) *+* (0 * 99)) / 361 = 6.2 % 90 / 361 = 0.249

**Pathological gaming**

Bisexual men (1 + (1 / (1 + 3) * 4) + (0 * 3)) / 23 = 8.7 % 4 / 23 = 0.173

Heterosexual men (5 + (5 / (5 + 18) * 40) + (0.9 * 99)) / 361 = 4.0 % 40 / 361 = 0.110

Mean 0.159

Min-Max 0.028; 0.312
